# High throughput phenotyping for aphid resistance in large plant collections

**DOI:** 10.1186/1746-4811-8-33

**Published:** 2012-08-17

**Authors:** Xi Chen, Ben Vosman, Richard GF Visser, René AA van der Vlugt, Colette Broekgaarden

**Affiliations:** 1 Wageningen UR, Plant Breeding, PO. Box 386, 6700, AJ, Wageningen, the Netherlands; 2 Plant Research International, PO Box 16, 6700, AA, Wageningen, the Netherlands

**Keywords:** Phloem-feeding insect, *Myzus persicae*, *Turnip yellows virus*, *Arabidopsis thaliana*, Activation tag

## Abstract

**Background:**

Phloem-feeding insects are among the most devastating pests worldwide. They not only cause damage by feeding from the phloem, thereby depleting the plant from photo-assimilates, but also by vectoring viruses. Until now, the main way to prevent such problems is the frequent use of insecticides. Applying resistant varieties would be a more environmental friendly and sustainable solution. For this, resistant sources need to be identified first. Up to now there were no methods suitable for high throughput phenotyping of plant germplasm to identify sources of resistance towards phloem-feeding insects.

**Results:**

In this paper we present a high throughput screening system to identify plants with an increased resistance against aphids. Its versatility is demonstrated using an *Arabidopsis thaliana* activation tag mutant line collection. This system consists of the green peach aphid *Myzus persicae* (Sulzer) and the circulative virus *Turnip yellows virus* (TuYV). In an initial screening, with one plant representing one mutant line, 13 virus-free mutant lines were identified by ELISA. Using seeds produced from these lines, the putative candidates were re-evaluated and characterized, resulting in nine lines with increased resistance towards the aphid.

**Conclusions:**

This *M. persicae*-TuYV screening system is an efficient, reliable and quick procedure to identify among thousands of mutated lines those resistant to aphids. In our study, nine mutant lines with increased resistance against the aphid were selected among 5160 mutant lines in just 5 months by one person. The system can be extended to other phloem-feeding insects and circulative viruses to identify insect resistant sources from several collections, including for example genebanks and artificially prepared mutant collections.

## Background

Phloem-feeding insects are among the most devastating pests worldwide, not only because of the direct damage caused by feeding, but also because of the viruses that many of them transmit. Viruses may be transmitted in a non-circulative or circulative way. In case of non-circulative viruses, like the potyviruses, the insect acquires the virus after a brief probe in an epidermal cell of a virus-infected plant. Subsequent probing on other (healthy) plants will transmit the virus from the aphids’ stylet to the plants [1]. Conversely, viruses that are transmitted in a circulative way, like members of the *Luteoviridae* family, are located in the phloem of the plant and insects can only acquire the virus by feeding for a prolonged period of time (up to 24 hours) from the phloem sap of infected plants [2]. The virus particles, taken up together with the phloem sap during feeding, cross the epithelial cells to diffuse through the haemolymph, and to finally be transported through the accessory salivary gland cells into the saliva and into a new plant during a subsequent feeding [3]. Once acquired, the virus can be maintained in the insect during the rest of its life. The efficiency of virus transmission is affected by plant traits conferring resistance against the vector insect. For instance, mechanical barriers may interfere with the insect’s ability to reach the phloem and subsequently reduce the transmission of virions [4].

Most phloem-feeding insects are able to transmit more than a 100 different plant viruses [2,5]. Due to genomic variation and high mutation rate, it is relatively easy for plant viruses to overcome the resistance of plants [6,7]. Therefore, it becomes an attractive strategy to search for resistance against the vector insect rather than for the resistance against each individual virus. At present, the main way to control phloem-feeding insects is via the frequent use of insecticides, which is only partly successful and hazardous to the environment. A more sustainable solution would be the use of plant varieties that are resistant to the insect. To be able to develop such resistant varieties, it is of utmost importance to identify resistant sources by screening plant collections, including genebank accessions or varieties, landraces and crop wild relatives, natural populations or even mutant collections [8-11]. In laboratory or green house experiments, plant resistance is normally quantified by using intact plants, detached leaves or even leaf disks to determine insect preference, population growth, survival and/or fecundity [12-16]. In field experiments insect resistance is usually measured by monitoring natural infestation levels [17]. These commonly used techniques are very time consuming due to the need of regular observations and tedious counting. Therefore, only relatively small collections have been screened for insect resistance so far, which seriously reduces the chance of identifying new resistant sources.

Here, we present a method that allows the screening of large plant collections for resistance towards phloem-feeding insects, using a circulative virus as indicator. We demonstrate the versatility of the method by screening a collection of *Arabidopsis thaliana* mutant lines [18] for increased resistance towards the aphid *Myzus persicae* using the *Turnip yellows virus* (TuYV) as an indicator. These mutant lines harbour a randomly inserted transposon bearing the *Cauliflower mosaic virus* (CaMV) 35S promoter [18]. Expression of genes located adjacent to the transposon may be increased leading to a gain-of-function phenotype [18]. The different mutated lines were inoculated using viruliferous aphids and plants escaping infection were looked for. Because this virus does not show any symptoms on *A. thaliana*, we performed double antibody sandwich enzyme-linked immunosorbent assays (DAS-ELISA) to detect infected plants. This aphid-virus system enabled a single person to phenotype 5160 *A. thaliana* mutant lines in five months and to identify nine mutant lines with increased aphid resistance.

## Results

### Selection and re-evaluation of aphid resistant candidates by the *M. persicae*-TuYV system

A total of 5160 mutant lines of *A. thaliana* were evaluated in four batches. Four viruliferous aphids were released on each plant for virus transmission and one plant per mutant line was tested. Leaf samples from 1280 mutant lines in the first batch were examined for TuYV infection by ELISA at 14 and 21 days post infestation (dpi) as TuYV does not show any symptoms on *A. thaliana*. This revealed that 99.9% of the mutant lines were infected at 14 dpi, i.e. one mutant line (4619) showed negative ELISA values whereas all others were positive, and 100% of them were infected at 21 dpi. To increase the chances of finding candidate mutants that may express partial increased resistance to aphids, the remainder of the mutant lines were tested at 14 dpi and 13 mutant lines were negative when assayed by ELISA result, indicating no or a very low virus concentration. To confirm the absence of virus infection of the 13 mutant lines, seeds were generated from these lines by selfing and 30 plants per mutant line were re-evaluated using the *M. persicae*-TuYV system. For nine mutant lines, a fraction of the plants showed a negative ELISA result, indicating that the virus was absent. Per mutant line tested the percentage of non-infected plants varied from 3.3% to 20% depending of the mutant line (Table 1). The remaining four mutant lines behaved like the wild type plants showing 100% of infection (Table 1).

**Table 1:** Frequency of non-infected plants and infection level in mutant lines and wild type

**Mutant lines**	**Frequency of non-infected plants (%)**	**Number of plants analysed**	**Mean OD ± SD**
Wild type plants	0	30	0.44 ± 0.13
807	0	22	0.41 ± 0.12
1912	0	30	0.37 ± 0.14
402	0	27	0.33 ± 0.12
1264	0	25	0.38 ± 0.14
1348	3.3	30	0.40 ± 0.14
3537	3.3	30	0.56 ± 0.16
3646	4.8	21	0.51 ± 0.12
3732	6.7	30	0.36 ± 0.15
2018	8.7	23	0.39 ± 0.09
3790	10	30	0.30 ± 0.12
4619	17	30	0.49 ± 0.21
3474	17	30	0.34 ± 0.11
1378	20	30	0.37 ± 0.15

Seeds were generated from selfed candidate mutant lines. Around 30 plants per mutant line were re-evaluated in *M. persicae*-TuYV system as described in “Methods”. ELISA values were optical density (OD) means ± SD of infected plants, with 0.073 ± 0.003 for non-inoculated plants.

### Characterization of the candidate mutant lines by aphid assays

**Figure 1:**
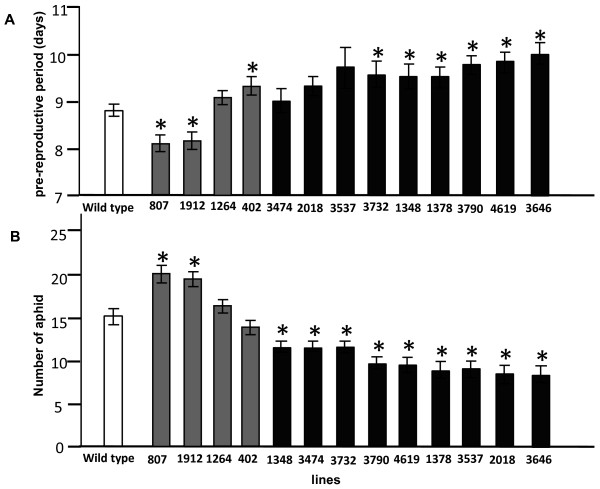
**Aphid performance on mutant lines and wild type.** Synchronized one-day-old nymphs were used to infest three-week-old *A. thaliana* plants with one nymph per plant. The time that a nymph began to reproduce was recorded (**A**). The total number of aphids was counted at 14 dpi (**B**) Values are the means ± SE of at least 16 plants. The asterisks indicate a significant difference compared to the wild type plants (*p* < 0.05, Independent-samples *t*-test).

The absence or the low viral infection of the selected mutated lines can be explained by a resistance of the plant to the virus or to the aphid. In order to discriminate between these two possibilities, aphid performance on the candidate line was followed. We monitored the pre-reproductive period and the population development of synchronized one day old nymphs. Aphid behaviour was negatively affected on the nine mutant lines for which a certain percentage of virus free plants were found in the re-evaluation of the aphid-virus system (Figure 1). Six mutant lines showed a delayed time to reproduction compared to the wild type, ranging from 0.5 to 1 day (Figure 1A). Aphid population size 14 dpi was significantly lower, up to 40% less, on all these nine mutant lines compared to the wild type (Figure 1B).

We also included in our analysis the four mutant lines that were initially identified by the aphid-virus system as candidates, but showed to be false negatives after re-assessment of the progeny, as all the plants of these mutant lines were infected by the virus in the re-evaluation. On two mutant lines (807 and 1912) the nymphs began to reproduce one day earlier than the nymphs on the wild type plants (Figure 1A). Subsequently, those two lines contained significantly more aphids than the wild type plants at 14 dpi (Figure 1B). For mutant line 1264 both the nymph development and the population development were comparable to that of the wild type plants; whereas the time to reproduce on mutant line 402 was slightly delayed but aphid population reached the same level as the one on wild type plants at 14 dpi (Figure 1).

## Discussion

### The aphid-virus system

In this paper we present an aphid-virus system that allows the screening for aphid resistance of a large collection of plants. Its versatility is demonstrated using an *A. thaliana* activation tag mutant collection [18]. In total 5160 mutant lines were tested using this system -by one person in five months-, resulting in the identification of nine mutant lines showing an increased level of resistance towards aphids. Previously, another *A. thaliana* activation tag mutant collection has been phenotyped for altered glucosinolate content after which the candidate lines were evaluated for resistance towards *M. persicae*[19,20]. This resulted in the identification of only one aphid resistant mutant line (*IQD*1) out of 16500 [19,20]. This targeted approach, i.e. selecting candidate lines based on altered glucosinolate content and then characterizing the lines with increased levels of glucosinolates for insect resistance, may explain the relative low number of insect resistant mutant lines identified in that study. To our knowledge, this aphid-virus system is the first method adapted to screen large collections of plants for resistance to phloem-feeding insects in an untargeted way. Using this approach many genes affecting the level of aphid resistance can be identified. The success in narrowing down the number of putative candidates was attributed to the use of TuYV, a circulative virus that can only be efficiently transmitted during phloem ingestion by the aphid. Certain plant traits may affect the aphid’s feeding behaviour and consequently the possibility and efficiency of virus transmission into plants. For instance, probing capability of the whitefly *Bemisia tabaci* has been shown to be reduced on tomato plants with acylsucrose-secreting type IV trichomes that consequently reduced the spread of *Tomato yellow leaf curl virus*[4].

The *M. persicae*-TuYV system can be easily used to screen large collections of plants in comparison to other time consuming and labour intensive methods that are used for identifying aphid resistant sources. For example, the arena setup is a frequently used method in which aphids are released in the middle of a circle formed by different plants and are allowed to choose a plant to feed on for a certain time period after which the number of aphids on each plant is counted [16]. Another commonly used method is based on non-choice tests in which aphids are confined to a plant or a specific leaf area by insect-proof cages and let to produce offspring [13,21]. For all these methods, regular monitoring and counting of aphid numbers is required to compare the insect preference/performance between plants which limits their applicability for screening large collections. When using the *M. persicae*-TuYV system thousands of plants can be grown at one time and tedious counting work is not required. The screening system holds the middle between a choice and non-choice assay, i.e. aphids and nymphs are transferred directly onto each plant, but the aphids/nymphs can move freely to other plants. This means that attraction/repellence, which can be influenced by the virus [22,23], may increase or decrease the number of aphids on a plant thereby affecting the outcome of the assay. This may result in the identification of false aphid resistant candidates in case aphids leave a susceptible plant due to repellence.

Plant traits that negatively affect aphid feeding behaviour may affect the timing of virus transmission and/or the number of virions that will be transferred into the phloem. Therefore we hypothesized that, compared to wild type plants, mutant plants expressing aphid resistance traits are infected at a later stage or with fewer virus particles resulting in a longer time for the virus to develop and, as a consequence, decrease the chance of detecting the virus after a certain time of infestation. This hypothesis is supported by the observation that the selected aphid resistant candidate mutants were not infected at 14 dpi whereas they all showed detectable virus levels at 21 dpi. Applying a shorter time for virus development, e.g. 7 dpi, may increase the number of aphid resistant candidates, but it may come with the disadvantage of more false candidates as well. Therefore, it should be noted that the high throughput of our method trades off with a relative high chance of overlooking candidates with increased resistance to aphids. Detection of the virus in plants from candidate mutant lines with increased aphid resistance in the re-evaluation can also be explained by our hypothesis that mutant lines expressing aphid resistance traits have a lower chance of detecting the virus at 14dpi compared to wild type plants as the percentage of non-infected plants was higher for these lines (3.3 to 20%) compared to the wild type (100%; Table 1). This also suggests that more aphid resistant mutant lines are present in the activation tag mutant collection but have not been identified in the initial screening.

As our method included one plant per mutant line only, there is a risk of missing aphid resistant candidates in the *Arabidopsis* activation tag library that we used due to the heterogeneity of some mutant lines, revealed by the absence of the BASTA resistance gene (the selectable marker present on the transposon). Obviously, this limitation can be overcome by testing more plants per mutant line.

### Mutant lines selected

All candidate mutant lines showed a reduced population development with non-viruliferous aphids (Figure 1B), indicating that plants with partial resistance to the aphid can be selected using our method. *Arabidopsis thaliana* is a suitable host to *M. persicae* and to our knowledge no accessions or mutant lines expressing a complete resistance to this aphid have been reported [24]. Therefore, it is not surprising that no *A. thaliana* mutant lines with full resistance against aphids have been identified in our study. Available literature shows differences in susceptibility levels only [25-27] that are comparable to the differences in population development between our mutant lines and the wild type. Part of the reduction in aphid population development on our candidate lines may be explained by a longer pre-reproductive period, but this is not the case for mutant lines 3474, 2018 and 3537. On these three lines, nymphs developed into adults similarly as on the wild type plants, suggesting that the increased resistance of the plant mainly affected the fecundity of the aphids.

Surprisingly, on two mutant lines that were initially selected but were found to be false negatives in the confirmation screen aphids showed a shorter pre-reproductive period and a larger population size than on wild type plants (Figure 1), meaning these two lines are better hosts to the aphids than wild type plants. This was completely contradictory to our expectations since this system was expected to identify mutant lines with a reduced aphid performance. So far we do not have any explanation for this unexpected finding.

Aphids have been widely used to study virus transmission and the mechanisms of plant resistance to virus [28,29]. However, it has been reported that the identified plant resistance to virus may actually be due to resistance against the vector aphids. For example, resistance to *Barley yellow dwarf virus* in some *Arogyron* species was due to the inability of aphid to reach the phloem [30]. In our screening we did not find any virus resistant mutant lines and probably more lines need to be tested to identify such resistance.

### Application

We have provided proof-of-concept for the versatility of the aphid-virus system using an *A. thaliana* activation tag mutant collection and the aphid *M. persicae.* Since *M. persicae* is not the only phloem-feeding insect that can vector plant viruses, our system can also be transposed to other phloem-feeding insects and circulative viruses as well as to other plant collections, i.e. other mutant libraries or genebank collections containing crops or crop wild relatives. For instance, the system may be used to identify plants with increased resistance to the whitefly *B. tabaci* using a geminivirus or the *Lettuce infectious yellows virus* as an indicator [31,32]. Similarly, resistance to corn planthopper *Graminella nigrifrons* and *Peregrinus maidis* may be identified with *Maize chlorotic dwarf virus* and *Sorghum stripe virus* as indicator respectively [33,34]. In addition to plant viruses, phytoplasmas are mainly transmitted by leafhoppers and psyllids that are also phloem-feeding insects [35]. Similar to the circulative plant virus, the phytoplasmas are taken up by the insect during phloem ingestion on an infected plant, cross the insect gut, amplify in the haemolymph, and circulate into the salivary glands. Then, the insect transfers the phytoplasmas to any plant when feeding [36]. Therefore, our insect-virus system could be applied in such combination for which circulative phytoplasmas may serve as an indicator for plant resistance against leafhoppers and psyllids.

We had used ELISA to detect the virus as it does not show any symptoms on *A. thaliana*. However, in a lot of cases one can use the virus symptoms as an indicator and thus circumvent the ELISA test. For instance, *Cucumber mosaic virus* infected tomato shows the deformation of leaves with stunted growth [37]; *Tomato yellow leaf curl virus* causes clear yellowing and curling symptoms on plant leaves [38], and *Potato virus Y* causes necrosis on potato leaves [39]. When a virus does not show any symptoms one may also consider developing an engineered virus that will induce symptoms development or adding the gene for the production of green fluorescent protein (GFP) [40] to the virus to visualize the presence of the virus in the plant. When a virus shows an asymptomatic infection or when symptoms can be induced by nutrient deficiencies [41] then molecular techniques such as quantitative RT-PCR can be used to detect the virus [42].

## Conclusions

In this paper we present a high-throughput phenotyping system, in which TuYV serves as an indicator for *M. persicae* resistance in *A. thaliana* plants. This aphid-virus system is a reliable method to identify candidates with increased resistance in a large plant collection. During the screening of 5160 mutant lines, nine lines with increased aphid resistance were identified. The aphid-virus system may be developed for other insect-virus combinations.

## Methods

### Aphids, plants and virus

*Myzus persicae*[43] was reared in cages on Chinese cabbage (*Brassica campestris* L. ssp. *pekinensis* var. Granaat). The rearing was maintained in an environment controlled room with a relative humidity of 60-70%. The temperature was set to 20 ± 2°C with an 18:6 L:D photoperiod. For all experiments, only apterous aphids were used.

A total of 5160 T-DNA activation-tag mutant lines of the *A. thaliana* accession Wassilewskija (WS) were obtained from the library present at Wageningen UR plant breeding [18]. Plants were cultivated in a climate chamber, programmed for a 6:18 L: D photoperiod. The temperature was maintained at 20 ±2°C during the day, 18 ±2°C during the night. The relative humidity was kept at 60-70%. Plants were grown on rockwool and supplemented with Hyponex nutrition solution every two days [44]. Three-week-old plants were used for all experiments. For seed collection plants were transferred, with the rockwool attached, into soil and placed in a greenhouse compartment at 20–22°C with an 18:6 L:D photoperiod and a relative humidity of 60-70%.

*Turnip yellows virus (*TuYV; family *Luteoviridae*, genus *Polerovirus)* was kindly provided by Dr. Véronique Brault of INRA Colmar, France. The virus was maintained on *Physalis floridana* plants that were kept in a cage located in the same growth chamber as the *A. thaliana* plants.

### Plant infestation/virus transmission

Aphids were collected from Chinese cabbage and released on detached leaves of TuYV infected *Physalis* plants [28] and allowed to feed for 48 hours [45] to obtain TuYV-viruliferous aphids. We used nymphs and adults to maximize the chances for successful TuYV transmission in our screening [46,47]. Two first- and second-instar nymphs together with two other third- and fourth-instar nymphs were transferred onto each *A. thaliana* plant using a fine brush. At 5 dpi, aphids were eliminated by applying 2 ml per plant of systemic insecticide, Admire, (0.05 gram/l; Bayer Cropscience) onto the rockwool.

### Virus detection by Double Antibody Sandwich-Enzyme linked immunosorbent assay

Because TuYV does not show any symptoms on *A. thaliana*, we conducted DAS-ELISA to detect the virus in plants. Two weeks post infestation with TuYV-viruliferous aphids two samples of newly developed leaves (approximately two square centimetres) were collected from each plant for the ELISA test. After leaf sample collection, plants were sprayed with BASTA (1 ml/li; Bayer Cropscience) to eliminate mutant lines without transposon insertion [18]. Only data from plants surviving the BASTA treatment were taken into account for further analysis. Leaf samples were kept in tubes (Corning, product #4408), which were filled with two metal balls (Ø 2 mm) and 200 μl of extraction buffer (0.01 M Phosphate Buffered Saline, pH 7.4, containing 1 ml/l Tween 20, 20 g/l of polyvinyl pyrrolidone and 2 g/l ovalbumine, grade VI). Plant tissue was grinded by using Retsch (American Instrument Exchange, 3519 N MILL) at a frequency of 30 cycles/second for one minute. One hundred μl plant extraction was analyzed by DAS-ELISA in immuno plates (Corning, product #9018) essentially as described by Clark and Adams in [48]. Previous to the ELISA procedure plates were coated o/n at 4°C with 100 μl 1:1000 (v/v) dilution in coating buffer (1.59 gr Na2CO3, 2.94 gr NaHCO3, 0.5 gr NaN3, pH 9.6/litre of coating antibodies against *Beet western yellows virus* (BWYV; the old name for TuYV). Antibodies were obtained from Prime Diagnotics (http://www.primediagnostics.com). Following incubation o/n at 4°C and washing plates were incubated for 3 hours at 37°C with 100 μl 1:1000 (v/v) dilution of Alkaline phosphatase conjugated BWYV antibodies (http://www.primediagnostics.com). After a final wash, the immuno plates were incubated with substrate (0.75 mg paranitrophenylphosphate (pNPP) in 97 ml/l of diethanolamine, pH 9.8) at room temperature for half an hour. The absorbance value (A405 nm) was measured in Model 680 Microplate Reader (Bio-Rad Laboratories (UK)) (Bio-RAD Model 680XR). To establish a threshold value for healthy plants, each immuno plate also contained eight samples of non-inoculated *A. thaliana* wild type plants. The absorbance values of these healthy samples were used to calculate a threshold for each plate, which was the average healthy value plus three times their standard deviation. Plant samples with absorbance values higher than the threshold were considered positive for infection with the virus.

### Aphid performance assay

To determine whether the candidate lines selected by the *M. persicae*-TuYV screening system were indeed aphid resistant mutant lines, we performed aphid assay in which the nymph pre-reproductive period and the population development on the candidate mutant lines were compared to those on wild type plants. Synchronized one-day-old nymphs were used to infest three-week-old *A. thaliana* plants with one nymph per plant. For the pre-reproductive period, the aphids were monitored twice a day at nine in the morning and at three in the afternoon from 6 till 12 dpi onwards. The time that a nymph began to reproduce was recorded. For the population development, the total number of aphids was counted at 14 dpi. After aphid number determination, plants in mutant lines were sprayed with BASTA to remove plants without transposon insertion. There was a minimum of 16 plants for each candidate mutant line as well as for wild type plants. Comparisons for aphid performance between mutant lines and wild type were analysed by independent-samples t-tests. *p* < 0.05 was used to detect statistical differences.

## Abbreviations

TuYV: *Turnip yellows virus*; BWYV: *Beet western yellows virus*; CaMV: *Cauliflower mosaic virus*; DAS-ELISA: Double antibody sandwich enzyme-linked immunosorbent assays; WS: Wassilewskija; pNPP: Paranitrophenylphosphate; GFP: Green fluorescent protein; OD: Optical density.

## Competing interests

The authors declare that they have no competing interests.

## Authors’ contributions

XC carried out the establishment of the screening system, conducted ELISA detection, aphid assay, performed the statistical analysis and drafted the manuscript. RGFV participated in drafting the manuscript. RAAV participated in experimental designs and coordination and helped to draft the manuscript. CB and BV conceived the study, participated in the design and participated in drafting the manuscript. All authors read and approved the final manuscript.
